# Editorial: COVID-19 and hyper inflammation syndrome: Different presentation and management

**DOI:** 10.3389/fped.2022.1022701

**Published:** 2022-09-09

**Authors:** Dimitri Poddighe, Vahid Ziaee, Ozgur Kasapcopur

**Affiliations:** ^1^Department of Medicine, Nazarbayev University School of Medicine, Nur-Sultan, Kazakhstan; ^2^Clinical Academic Department of Pediatrics, National Research Center for Maternal and Child Health, University Medical Center (UMC), Nur-Sultan, Kazakhstan; ^3^Department of Pediatrics, Tehran University of Medical Sciences, Tehran, Iran; ^4^Children's Medical Center, Pediatric Center of Excellence, Tehran, Iran; ^5^Department of Pediatric Rheumatology, Istanbul University-Cerrahpasa, Istanbul, Turkey

**Keywords:** COVID-19, SARS-CoV-2, children, MIS-C, Kawasaki disease (KD)

More than 2 years after the declaration of a pandemic for SARS-CoV-2 infection, COVID-19 is still a major public health, social and economic issue worldwide (1). Although children are less severely affected by SARS-CoV-2 infection than adults, during these years the impact of COVID-19 on the pediatric population has clearly emerged in all its facets, which were not entirely evident at the beginning of the pandemic (2, 3).

Most clinical studies were carried out in developed countries, but pediatric COVID-19 represents a relevant problem even in developing countries, where performing well-designed clinical studies may be more difficult (4–6). The articles included in this Research Topic are from different continents (Europe, Asia, and America) and investigated several aspects of the clinical presentation, pathophysiological mechanisms, and medical management of SARS-CoV-2 infection in children.

Through their case reports, Generalić et al., Emeršič et al., Artamonova et al., and Matsubara et al. emphasized how COVID-19 can also present with protean and unusual clinical manifestations in children.

However, the most challenging clinical aspect of pediatric SARS-CoV-2 infection is represented by the multisystem inflammatory syndrome in children (MIS-C). In their article submitted in the first part of 2021, Matucci-Cerinic et al. analyzed MIS-C by comparing it with Kawasaki Disease (KD). They listed the main similarities and differences between these hyper-inflammatory disorders and hypothesized that MIS-C could be viewed as a disorder included in the KD spectrum, instead of representing a completely new inflammatory disorder of childhood. In detail, they suggested that the development of KD or MIS-C phenotypes during or after SARS-CoV-2 infection might depend on several factors, including (but not limited to) viral load, virulence of SARS-CoV-2 strain(s), child's age, intensity/kinetics of the immune response, ethnic/genetic background, and comorbidities. Additional research seemed to support this view that some common pathophysiological patterns are shared by KD and MIS-C. For instance, Ghosh et al. (7) evidenced some similar cytokine patterns and, in general, host immune responses in MIS-C and KD. From the clinical side, Yilmaz Ciftdogan et al. (8) analyzed the characteristics of MIS-C in 614 children with and without overlap with KD: they reported that almost half patients with MIS-C had clinical features overlapping with KD and, in particular, incomplete forms.

However, even though MIS-C and KD may be part of the same clinical and pathological spectrum and share some immunological mechanisms, these two entities differ by age of presentation and other clinical/immuno-genetic aspects, of course, as emphasized by Dhaliwal et al. These authors also stressed the concerns regarding the specific long-term cardiovascular sequalae of MIS-C, since these children present with acute myocardial injury/myocarditis. In this regard, Mamishi et al. also discussed the importance of the myocardial systolic evaluation in children affected with COVID-19, even in patients without MIS-C.

Therefore, although MIS-C and KD may be included in the same immuno-pathological spectrum, there are clinical and prognostic differences, which require these two entities to be timely and clearly diagnosed and differentiated, in order to grant patients with the most appropriate clinical management (9). The study by Kostic et al. aimed to create a Kawasaki/MIS-C differentiation score (KMDscore) for the discrimination between these two diseases. Indeed, compared with COVID-19 in general and KD, patients with MIS-C may have significantly higher prevalence of cardiac complications and more elevated markers of inflammation and cardiac damage: therefore, diagnostic scores could be a useful tool for distinguishing MIS-C from KD and, thus, should be a priority for clinical research (10).

In addition to a prompt and precise diagnosis of MIS-C, the appropriate medical care and treatment is the other fundamental point for a successful outcome in these sick children (11). In this special collection, several groups reported their clinical experiences. Menchaca-Aguayo et al. described 90 Mexican patients diagnosed with pediatric inflammatory multisystem syndrome, temporally associated with “SARS-CoV-2 (PIMS-TS)/multisystem inflammatory syndrome in children (MIS-C).” They reported a good clinical outcome with null mortality by treating their patients with corticosteroids, alone or combined with intra-venous immunoglobulin (IVIG). They also described those factors resulting more significantly associated with pediatric intensive care unit admission in their center, which were older age, shock at admission, and hypoalbuminemia. According to their initial experience with MIS-C patients in Italy, Brisca et al. proposed a multistep anti-inflammatory treatment protocol for MIS-C based on the “Gaslini severity assessment tool” for MIS-C, which differentiates these patients in 4 classes eligible to progressively more intense treatments (class I: IVIG 2 g/kg; class II: IVIG 2 g/kg + methylprednisolone 2–3 mg/kg/day; class III: IVIG 2 g/kg + pulsed methylprednisolone 10–30 mg/kg/day; class IV: IVIG 2 g/kg + pulsed methylprednisolone 10–30 mg/kg/day + anakinra 5–10 mg/kg/day–max. 100 mg.). Licciardi et al. also supported the importance of a tailored step-up treatment (including IVIG, methylprednisolone and anakinra) of MIS-C for a more successful outcome. Recent (systematic) reviews and meta-analyses further supported the aforementioned therapeutic approach, in general (12–14).

In conclusion, all these research efforts from many countries have significantly contributed to increase the knowledge on pathophysiological, diagnostic, and therapeutic aspects of MIS-C and, in general, pediatric COVID-19 in the last 2 years.

## Author contributions

DP drafted and wrote the manuscript. OK and VZ reviewed the manuscript. All authors approved published version of the manuscript.

## Conflict of interest

The authors declare that the research was conducted in the absence of any commercial or financial relationships that could be construed as a potential conflict of interest. The handling editor ED declared a past co-authorship with the authors DP and OK.

## Publisher's note

All claims expressed in this article are solely those of the authors and do not necessarily represent those of their affiliated organizations, or those of the publisher, the editors and the reviewers. Any product that may be evaluated in this article, or claim that may be made by its manufacturer, is not guaranteed or endorsed by the publisher.
